# Cephalometric study to test the reliability of anteroposterior skeletal discrepancy indicators using the twin block appliance

**DOI:** 10.1186/s40510-015-0073-1

**Published:** 2015-02-25

**Authors:** Rahul Trivedi, Amit Bhattacharya, Falguni Mehta, Dolly Patel, Harshik Parekh, Vaibhav Gandhi

**Affiliations:** Department of Orthodontics, Government Dental College and Hospital, Asarva, Ahmedabad, 380016 Gujarat India; AMC Dental College, Ahmedabad, 380008 Gujarat India

**Keywords:** Anteroposterior discrepancy parameters, Twin block, Lateral cephalogram, Reliability

## Abstract

**Background:**

The objectives of this study were to check the reliability of the five angular and two linear parameters for sagittal maxillo-mandibular discrepancy and to compare and correlate angular parameters with the ANB angle, and the linear parameter with Wits analysis.

**Methods:**

The pre-treatment and post-functional lateral cephalograms of 25 subjects (17 males, 8 females) with class II division 1 malocclusion treated with twin block functional appliance were selected. Five angular (ANB, β angle, APDI, YEN angle, W angle) and two linear (Wits analysis, App-Bpp) parameters were traced on both sets of cephalograms. Paired Student’s *t*-test, one-way ANOVA, *post hoc* test, and Karl Pearson correlation statistical analysis were performed.

**Results:**

All the parameters considered in our study showed highly significant difference in pre-treatment and post-functional values, suggesting their reliability (*p* < 0.0001). When ANB angle was compared with the other angular parameters, a highly significant change in the mean value of the difference in pre-treatment (T1) and post-functional (T2) values was noted (*p* < 0.001). No significant change was seen when comparing the mean value of the difference in T1 and T2 between linear parameters (*p* = 0.949).

**Conclusions:**

All the parameters used in the study can be reliably used to assess anteroposterior skeletal discrepancy. Whenever limitations of the ANB angle and Wits analysis are foreseen, the W angle and App-Bpp, respectively, can be reliably used. The YEN angle may reliably predict the post-functional change with the use of twin block appliance.

## Background

Patients seeking orthodontic treatment frequently fall in the skeletal class II category. The National Health and Nutrition Examination Survey estimated, based on overjet, that approximately 14.7% of the US population has class II malocclusion, with prevalence decreasing from 22.6% between 8 and 11 years of age, to 15.6% between 12 and 17 years of age and then to 13.4% between 18 and 50 years of age [1]. The National Center for Health Statistics reported that 20.4% of 6- to 11-year-olds have bilateral class II molar relationships, compared with 14.5% of 12- to 17-year-olds [2,3]. The prevalence of skeletal class II type of malocclusion in the Indian population is 14.6% for the age group of 10 to 13 years, 6% for the age group of 5 to 9 years, and 3.8% for the age group of 6 to 14 years [4]. Although maxillary protrusion and mandibular retrognathism are both found to be possible causative factors, McNamara reported mandibular retrognathism to be more common for skeletal class II malocclusion [5].

Evaluation of anteroposterior jaw discrepancy for diagnosis and treatment planning is one of the primary requirements in the field of orthodontics. Numerous authors have tried to assess the sagittal skeletal relationship using various landmarks, starting from Riedel (ANB angle) [6], Jacobson (Wits analysis) [7], Nanda and Merrill (App-Bpp) [8], Baik and Ververidou (β angle) [9], Kim and Vietas (APDI) [10], Neela et al. (YEN angle) [11], Bhad (W angle) [12], etc. The ANB angle and Wits analysis are still one of the most commonly and reliably used parameters in assessing maxillo-mandibular relation, although there is not a single diagnostic test or cephalometric measurement that has been accepted to be used as the ‘gold standard’ for defining class II or class III skeletal patterns [13-16]. Accurate location of the cephalometric landmarks, growth changes, orthodontic treatment, etc. may influence the accurate assessment of the sagittal skeletal discrepancy. Limitations of these measurements add to the confusion regarding the reliability of these parameters to assess anteroposterior jaw discrepancy.

The treatment for class II malocclusion varies from skeletal growth modification to camouflage to orthognathic surgery depending upon the age of the patient. It is a well-known fact that the twin block appliance developed by Clark is an effective class II corrector in skeletal growth modification [17-19]. Although many studies have been carried out and documented showing anteroposterior skeletal assessment with twin block appliance, no study has been conducted comparing and correlating all the parameters of sagittal changes using twin block. So it was considered worthwhile to do quantitative appraisal of the reliability of five angular and two linear sagittal skeletal discrepancy parameters to assess the result of using twin block appliance for correction of class II malocclusion. Taking the above stated fact into consideration, it was decided to assess the change in the maxillo-mandibular relation using five angular (ANB angle, APDI, β angle, W angle, YEN angle) and two linear (Wits analysis and App-Bpp) anteroposterior discrepancy parameters in patients who were treated with the twin block appliance.

The objectives of the study thus were as follows:

- To check the reliability of the angular and linear parameters considered in our study
- To compare and correlate the angular parameters
- To compare and correlate the linear parameters
- To evaluate which of the above parameters best predicts the sagittal correction after twin block therapy

## Methods

The present cephalometric study was undertaken from the records available in the Department of Orthodontics Govt. Dental College and Hospital. Twenty-five cases (17 males, 8 females) in the age range of 11 to 14 years having CVMI in stages III and IV who fulfilled the following inclusion criteria were selected for the study:

1. Angle’s class II division 1 malocclusion as evaluated from the dental study casts
2. ANB angle greater than 4° with mandibular deficiency
3. Overjet ranging from 6 to 15 mm with a mean of 10 mm
4. No prior orthodontic treatment
5. Subjects treated with only removable twin block functional appliance

All subjects were given modified twin block appliance with jack screw and labial bow incorporated in the maxillary plate. They were regularly called for follow-up and evaluated for sagittal correction. After bilateral molar class I reduction in overjet and after significant improvement in profile was achieved, post-functional records were made. The average treatment time of twin block functional appliance therapy was 11 months.

Pre-treatment (T1) and post-functional (T2) lateral cephalograms were obtained and traced by a single operator. Five angular and two linear parameters were taken for both sets of cephalograms and were compared and evaluated.

The angular parameters used were as follows (Table 1):

**Table 1:** **Short description of various parameters used in the study**

**Parameter**	**Short description**
ANB	The angle formed by the intersection of the point A to N (nasion) line and the point B to N (nasion) line (Figure 1)
β angle	A perpendicular is dropped from point A to a line drawn from C (center of condyle) to point B. β angle is the angle between this perpendicular and the A-B line (Figure 2)
YEN angle	Angle between the S (sella) to M (center of the premaxilla) line and the M to D (center of the symphysis) line (Figure 3)
W angle	A perpendicular is dropped from M to the S-D line. W angle is measured between this perpendicular and the M-D line (Figure 4)
APDI	(NPg-FH plane) ± ( NPg-AB) ± (FH-palatal plane) (Figure 5)
Wits appraisal	Distance between the projection of point A and point B on the occlusal plane (Figure 6)
App-Bpp	Distance between the projection of point A and point B on the palatal plane (Figure 6)

**Figure 1:**
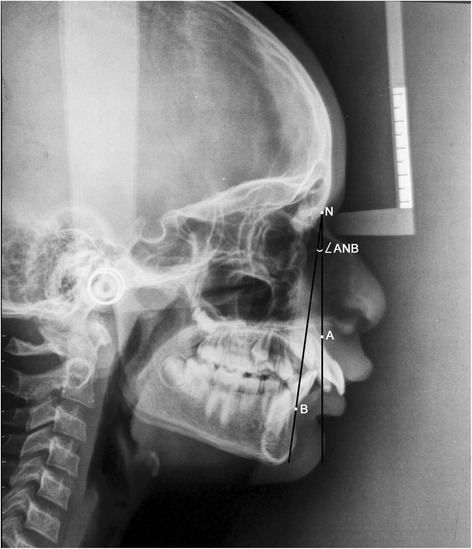
**Angular parameter ANB.**
- β angle (Figure 2)

- ANB angle (Figure 1)

**Figure 2:**
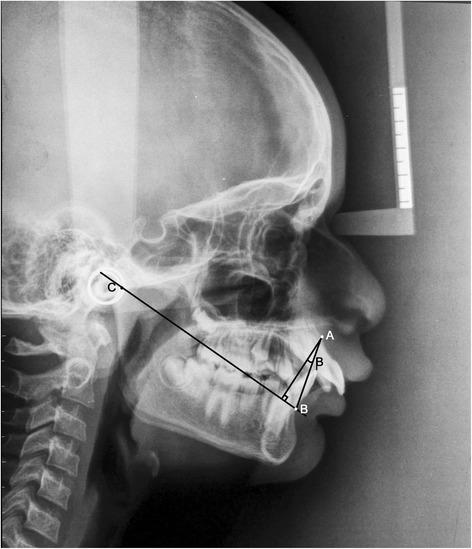
**Angular parameter β angle.**
- YEN angle (Figure 3)

**Figure 3:**
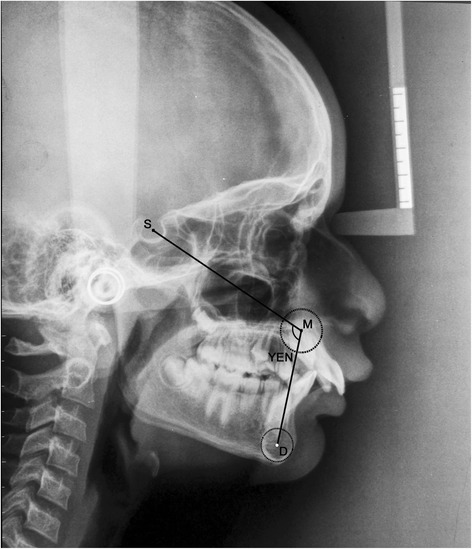
**Angular parameter YEN angle.**
- W angle (Figure 4)

**Figure 4:**
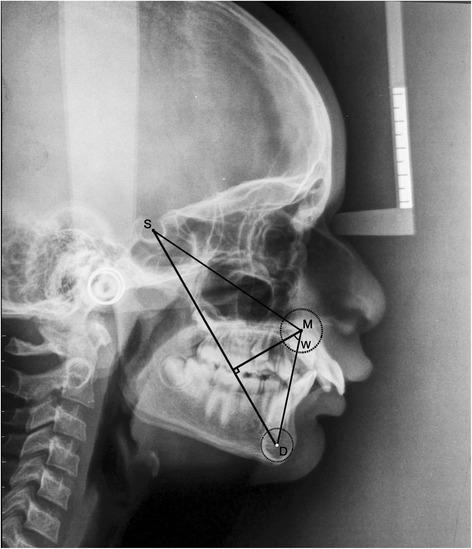
**Angular parameter W angle.**
- APDI (Figure 5)

**Figure 5:**
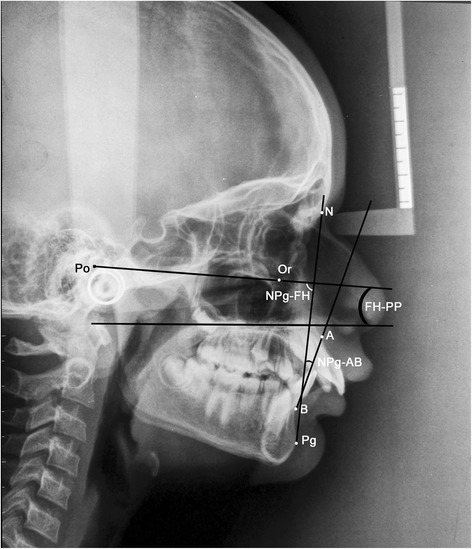
**Angular parameter APDI.**

**Figure 6:**
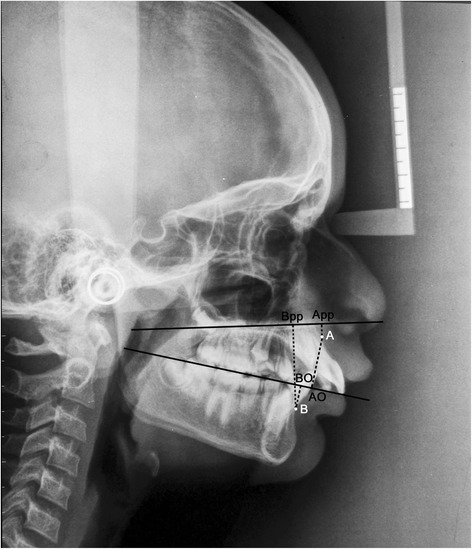
**Linear parameters App-Bpp and Wits appraisal.**

The linear parameters used were as follows (Figure 6, Table 1):

- Wits analysis
- App-Bpp

The resultant data was subjected to statistical analysis. Paired sample *t*-test was performed to evaluate the differences in the pre-treatment (T1) and post-functional (T2) values (Table 2) in the angular and linear parameters. ANOVA (Table 3) and Tukey HSD *post hoc* test (Table 4) for multiple comparisons were done to evaluate change in the mean of the difference between T1 and T2 values of the parameters. Correlations between angular parameters and linear parameters were assessed using the Karl Pearson correlation coefficient test (Table 5). The ability of a parameter to predict the probable change in the sagittal plane after treatment with the twin block appliance was evaluated using regression equations as well as the standard error of the estimate (Table 6). *r*, the multiple correlation coefficient, is the linear correlation between the observed and model-predicted values of the dependent variable. *r*^2^, the coefficient of determination, is the squared value of the multiple correlation coefficient. It shows that about half the variation in time is explained by the model.

**Table 2:** **Paired sample** ***t*** **-test comparing pre-treatment (T1) and post-functional (T2) values**

	**Mean**	***N***	**Standard deviation**	**Standard error of the mean**	**Mean difference**	***p*** **value**
ANB angle (T1)	7.36	25	1.469	0.294	−3.84	<0.0001*
ANB angle (T2)	3.52	25	1.388	0.278		
Wits appraisal (T1)	6.72	25	2.092	0.418	−5.46	<0.0001*
Wits appraisal (T2)	1.26	25	1.849	0.37		
β angle (T1)	19.12	25	3.153	0.631	8.56	<0.0001*
β angle (T2)	27.68	25	3.024	0.605		
APDI (T1)	70.2	25	3.64	0.728	7.76	<0.0001*
APDI (T2)	77.96	25	3.702	0.74		
App-Bpp (T1)	12.52	25	2.801	0.56	−4.72	<0.0001*
App-Bpp (T2)	7.8	25	2.179	0.436		
YEN angle (T1)	114.12	25	4.799	0.96	4.8	<0.0001*
YEN angle (T2)	118.92	25	5.227	1.045		
W angle (T1)	49.52	25	3.38	0.676	4.2	<0.0001*
W angle (T2)	53.72	25	3.361	0.672		

T1, pre-treatment; T2, post-functional; *p ≤ 0.05 = significant.

**Table 3:** **Analysis of variance test between the parameters**

**Parameter**	***N***	**Mean**		**Standard deviation**	**Standard error**
ANB angle	25	−3.84		1.028	0.206
Wits appraisal	25	−5.46		2.291	0.458
β angle	25	8.56		3.097	0.619
APDI	25	7.76		3.767	0.753
App-Bpp	25	−4.72		2.132	0.426
YEN angle	25	4.80		2.661	0.532
W angle	25	4.20		2.102	0.420
Total	175	1.61		6.194	0.468
	**Sum of squares**	***Df***	**Mean square**	***F***	**Overall ANOVA** ***p*** **value**
Difference between parameters	5,569.134	6	928.189	140.853	<0.0001*
Total	6,676.214	174			

*p ≤ 0.05 = significant.

**Table 4:** ***Post hoc*** **tests (Tukey HSD): multiple comparisons**

**Parameter**	**Parameter**	**Mean difference**	**Standard error**	***p*** **value**
ANB angle	β angle	−12.40	0.726	<0.0001*
ANB angle	APDI	−11.60	0.726	<0.0001*
ANB angle	YEN angle	−8.64	0.726	<0.0001*
ANB angle	W angle	−8.04	0.726	<0.0001*
Wits appraisal	App-Bpp	−0.74	0.726	0.949
β angle	APDI	0.80	0.726	0.927
β angle	YEN angle	3.76	0.726	<0.0001*
β angle	W angle	4.36	0.726	<0.0001*
APDI	YEN angle	2.96	0.726	0.001
APDI	W angle	3.56	0.726	<0.0001*
YEN angle	W angle	0.60	0.726	0.982

*p ≤ 0.05 = significant.

**Table 5:** **A correlation matrix for the seven parameters calculated with the Karl Pearson correlation test**

	**ANB angle**		**Wits appraisal**	**β angle**	**APDI**	**App-Bpp**	**YEN angle**	**W angle**
ANB angle	Pearson correlation (*r*)		0.563	−0.527	−0.420	0.530	−0.445	−0.613
	Significance (two-tailed) (*p*)		0.003*	0.007*	0.037*	0.006*	0.026*	0.001*
	*n*			25	25	25	25	25
Wits appraisal	Pearson correlation (*r*)			−0.552	−0.185	0.475	−0.217	−0.383
	Significance (two-tailed) (*p*)			0.004*	0.377	0.016*	0.297	0.059
	*n*				25	25	25	25
β angle	Pearson correlation (*r*)				0.455	−0.441	0.328	0.424
	Significance (two-tailed) (*p*)				0.022*	0.027*	0.110	0.035*
	*n*					25	25	25
APDI	Pearson correlation (*r*)					−0.552	0.593	0.591
	Significance (two-tailed) (*p*)					0.004*	0.002*	0.002*
	*n*						25	25
App-Bpp	Pearson correlation (*r*)						−0.548	−0.655
	Significance (two-tailed) (*p*)						0.005*	0.0004*
	*n*							25
YEN angle	Pearson correlation (*r*)							0.894
	Significance (two-tailed) (*p*)							<0.0001*
	*n*							25
W angle	Pearson correlation (*r*)							
	Significance (two-tailed) (*p*)							
	*n*							

*p ≤ 0.05 = significant.

**Table 6:** **Correlation coefficient and regression equation**

**Parameter**	***r***	***r*** ^**2**^	**Equation**	**Standard error of the estimate**
ANB angle	0.74	0.55	*Y* = 0.702*X − 1.645	0.95
Wits appraisal	0.33	0.11	*Y* = 0.291*X − 0.696	1.78
β angle	0.50	0.25	*Y* = 0.478*X + 18.549	2.68
APDI	0.47	0.22	*Y* = 0.482*X + 44.140	3.33
App-Bpp	0.66	0.43	*Y* = 0.513*X + 1.375	1.67
YEN angle	0.86	0.74	*Y* = 0.940*X + 11.697	2.70
W angle	0.81	0.65	*Y* = 0.801*X + 14.059	2.03

*r*, the multiple correlation coefficient; *r* ^2^, the coefficient of determination, is the squared value of the multiple correlation coefficient.

Approval for this study was obtained from the institutional review board of the Govt. Dental College and Hospital, Ahmedabad, with informed consents from the parents or guardians of all subjects.

## Results and discussion

Paired sample *t*-test results showed highly significant changes between T1 and T2 for all the seven anteroposterior discrepancy parameters (*p* < 0.0001) (Table 2).

The ANOVA test showed the variation in the mean of the difference between T1 and T2 of all seven parameters to be highly significant (*p* < 0.0001) (Table 3).

Furthermore, when the ANB angle was compared with the other four angular parameters, a highly significant change in the mean of the difference between T1 and T2 values was noted (Table 4).

No significant change was seen when comparing the mean of the difference between T1 and T2 of the linear parameters (*p* = 0.949) (Table 5).

The change in the mean of the difference between T1 and T2 was highly significant for the β angle and YEN angle, β angle and W angle, and APDI and W angle (*p* < 0.0001) and were non-significant for the β angle and APDI (*p* = 0.927), and YEN angle and W angle (*p* = 0.982) (Table 4).

The Karl Pearson correlation test showed moderately negative but significant correlations for the angular parameters when they were compared to the ANB angle (Table 4). The highest correlations of all angular parameters compared with the ANB were observed for the W angle (*r* = −0.613). Moderately positive and significant correlation between App-Bpp and Wits analysis was also noted (Table 5). Strongly positive and highly significant correlations were observed only between the YEN angle and W angle (*r* = 0.894, *p* < 0.0001) (Table 5).

Regression equation analysis calculated for the YEN angle (*Y* = 0.940*X + 11.697) had the highest correlation coefficient (*r* = 0.86, *r*^2^ = 0.74). The standard error of the estimate was found to be least for ANB (0.95) (Table 6).

In our study, changes in the anteroposterior plane were assessed in the same group of patients after giving them a functional appliance, thus removing the subject error. The ultimate goals of this study were to assess the reliability of five angular and two linear sagittal skeletal discrepancy parameters and to compare and correlate the angular parameters (β angle, APDI, W angle, and YEN angle) with the universally accepted ANB angle, and the linear parameter App-Bpp with Wits analysis, as indicators of successful twin block therapy in growing subjects.

The primary objective to use twin block appliance was because it is a proven potent class II corrector [17-19]. Table 2 shows that there is a highly significant change in the pre-treatment (T1) and post-functional (T2) values of all the seven parameters considered in our study (*p* = <0.0001), suggesting that the sagittal change produced by the twin block is assessed accurately by all the seven parameters, thus confirming that any of the above parameters can be used reliably to assess anteroposterior discrepancy.

One of the oldest and widely used parameter is the ANB angle [6]; however, the stability of the nasion point is questionable as shown in growth studies by Nanda [20]. The rotation of the head sideways or upwards, rotation of the jaws either due to growth or orthodontic treatment, and rotation of the S-N plane also can affect the value of the ANB angle. Position of point A is affected by alveolar bone remodeling associated with orthodontic tooth movement of the upper incisor. Binder proposed that even the change in the vertical distance between points A and B without any change in the sagittal position may affect the ANB angle [21]. But the results of recent studies indicate that the ANB angle is still a reliable indicator to assess anteroposterior skeletal change before and after treatment, in spite of its limitation in diagnosis [22]. According to Table 3, 4, and 5, results showed that all the angular parameters vary independent of the ANB angle. Thus, it is suggested that when the abovementioned limitations of the ANB angle are anticipated, any of the other angular parameters such as the β angle (mean difference = −12.40, *r* = −0.527), APDI (mean difference = −11.60, *r* = −0.420), W angle (mean difference = −8.04, *r* = −0.613), YEN angle (mean difference = −8.64, *r* = −0.445), and especially W angle can be used to assess anteroposterior skeletal discrepancy as the limitations of the ANB angle are less likely to influence their values. Ishikawa et al. in their study found similar values for ANB and APDI [23]. Among the angular parameters considered, the W angle showed the highest correlation with the ANB angle (mean difference = −8.04, *p* = < 0.0001, *r* = −0.613).

Owing to the limitations of ANB, Jacobson came up with the Wits analysis [7]. Comparison of the linear parameters (Wits analysis and App-Bpp) from Tables 4 and 5 suggests that they do not vary independent of each other (mean difference = −0.74, *p* = 0.949, *r* = 0.475) which indicates a non-significant correlation between Wits and App-Bpp. Therefore, in cases where Wits analysis does not accurately depict the maxillo-mandibular relationship, App-Bpp also may give erroneous readings. However, since Wits analysis utilizes the occlusal plane, the value can be easily affected due to orthodontic treatment and incomplete tooth eruption. Accurate identification of the occlusal plane and its reproducibility in mixed dentition, open bite, canted occlusal plane, multiple impactions, and skeletal asymmetry subjects is difficult [24]. The palatal plane is considered to be more stable than the occlusal plane [8]. Thus, in cases where identification of anatomic landmarks required for Wits analysis is not clear, it is suggested to use App-Bpp in lieu of Wits analysis. However, when the palatal plane is severely tipped, App-Bpp may also give erroneous readings [8]. In such instances, Wits analysis can be used reliably.

According to Tables 4 and 5, it is suggested that either the YEN angle or W angle can be used to assess skeletal anteroposterior discrepancy (mean difference = −0.6, *p* = 0.982, *r* = 0.894). With reference to the YEN angle, jaw rotations due to growth and treatment can mask true skeletal dysplasia [25]. The YEN angle requires accurate tracing of the premaxilla for its assessment. The W angle also requires accurate tracing of the premaxilla and locating its center, which is difficult. Also, determining which of the jaws is prognathic or retrognathic is difficult [12]. Thus, when accurate tracing of the premaxilla for the assessment of the W angle and YEN angle is not possible, the β angle can be used as it shows least correlation with the YEN angle and W angle (Tables 4 and 5).

No significant change in the mean of the difference between T1 and T2 values of the β angle and APDI was found (mean difference = 0.8, *p* = 0.927, *r* = 0.455) (Tables 4 and 5). The β angle uses the condylion as a reference point, but its identification on a closed mouth lateral cephalogram is difficult [26-28]. Thus, APDI can be used in lieu of the β angle when locating the condylion is difficult.

The ability of the angular and linear parameters considered in our study to predict the amount of post-functional change in the maxillo-mandibular relation was analyzed with regression equations (Table 6), and it was found that the YEN angle may reliably predict post-functional change when twin block appliance is used. Strong predictability was also found for the W angle (*r* = 0.81, *r*^2^ = 0.65) and ANB angle (*r* = 0.74, *r*^2^ = 0.55). Although the ANB angle showed the least error (Table 6), it has to be kept in mind that the normal range in ANB for different classes of malocclusion is also relatively small. App-Bpp showed better predictability when compared to Wits analysis (Table 6). These results are in contrast to those of Ishikawa et al. for the parameters ANB, Wits, and APDI [23].

Whenever limitations of any of the parameters used in our study are expected to cloud the clinical judgment in assessing skeletal anteroposterior relationship, use of more than one parameter in conjunction with others as described in our study is suggested.

## Conclusions

Twenty-five subjects were treated with twin block appliance, and the change in their maxillo-mandibular relation was assessed with five angular and two linear parameters.

1. All the angular and linear parameters considered in our study are reliable in assessing skeletal sagittal discrepancy.
2. Recently advocated angular parameters, especially the W angle, can be used reliably in place of the ANB angle whenever drawbacks of the ANB angle are anticipated.
3. As the palatal plane is more stable than the occlusal plane, it is suggested to use App-Bpp instead of Wits analysis.
4. The YEN angle and W angle show the highest correlation among the parameters, which suggests that either of them can be used to assess anteroposterior jaw discrepancy.
5. Then YEN angle may reliably predict the post-functional change with the use of twin block appliance.

## Abbreviations

T1: Pre-treatment
T2: Post-functional
